# Design of a Multiple-Behavior Change Intervention for Supporting Self-management in Patients With Chronic Heart Failure: An Intervention Mapping Approach

**DOI:** 10.1097/JCN.0000000000001095

**Published:** 2024-03-15

**Authors:** Joëlle Dam, Thijs M. H. Eijsvogels, Marjolein H. I. Verdijk, Anna M. Janssen, Bram M. A. van Bakel, Lisette E. H. J. M. Baltussen, Gert P. Westert, Marijn de Bruin

**Affiliations:** **Joëlle Dam, MSc** PhD Student, Department of IQ Health, Radboud Institute for Health Sciences, Radboud University Medical Centre, Nijmegen, the Netherlands.; **Thijs M. H. Eijsvogels, PhD** Associate Professor, Department of Physiology, Radboud Institute for Health Sciences, Radboud University Medical Centre, Nijmegen, the Netherlands.; **Marjolein H. I. Verdijk, MSc** Nurse Specialist, Department of Cardiology, Radboud University Medical Centre, Nijmegen, the Netherlands.; **Anna M. Janssen, MSc** PhD Student, Department of IQ Health, Radboud Institute for Health Sciences, Radboud University Medical Centre, Nijmegen, the Netherlands.; **Bram M. A. van Bakel, PhD** PhD Student, Department of Physiology, Radboud Institute for Health Sciences, Radboud University Medical Centre, Nijmegen, the Netherlands.; **Lisette E. H. J. M. Baltussen, MSc** Nurse Specialist, Department of Cardiology, Radboud University Medical Centre, Nijmegen, the Netherlands.; **Gert P. Westert, PhD** Professor, Department of IQ Health, Radboud Institute for Health Sciences, Radboud University Medical Centre, Nijmegen, the Netherlands.; **Marijn de Bruin, PhD** Professor, Department of IQ Health, Radboud Institute for Health Sciences, Radboud University Medical Centre, Nijmegen, the Netherlands.

**Keywords:** counseling, heart failure, nursing, patient adherence, self-management

## Abstract

**Background:**

Nonadherence to medication and low physical activity contribute to morbidity, mortality, and decreased quality of life among patients with chronic heart failure (CHF). Effective interventions that can be delivered during routine clinical care are lacking.

**Objective:**

We aimed to adapt the feasible and cost-effective Adherence Improving self-Management Strategy (AIMS) for patients with human immunodeficiency virus (HIV) to CHF treatment. Subsequently, we determined its acceptability and feasibility.

**Methods:**

Adherence Improving self-Management Strategy is a systematic, nurse-delivered counseling intervention blended with eHealth to facilitate patient self-management. We used the intervention mapping framework to systematically adapt AIMS-HIV to AIMS-CHF, while preserving essential intervention elements. Therefore, we systematically consulted the scientific literature, patients with CHF and nurses, and pretested intervention materials.

**Results:**

Adherence Improving self-Management Strategy–HIV was modified to AIMS-CHF: a multiple-behavior change intervention, focused on medication adherence and physical activity. Key self-management determinants (such as attitudes, self-efficacy, and self-regulatory skills) and organization of care (such as specialized nurses delivering AIMS) were similar for HIV and heart failure care. The AIMS protocol, as well as material content and design, was systematically adapted to CHF. Preliminary testing suggests that AIMS-CHF is likely feasible and acceptable to patients with CHF and care providers.

**Conclusion:**

Using the intervention mapping protocol, AIMS-HIV could be systematically adapted to AIMS-CHF and seems acceptable and feasible. Evidence from the literature, behavioral theory, and input from nurses and patients were essential in this process. Adherence Improving self-Management Strategy–CHF should now be tested for feasibility and effectiveness in routine care.

Chronic heart failure (CHF) is a progressive and dynamic chronic disease, characterized by periods of stability interrupted by episodes of acute exacerbation. Exacerbations require urgent evaluation and treatment, often resulting in hospitalization.^1^ Although medical treatment improves CHF prognosis, the prognosis remains poor, with a high mortality rate and low quality of life.^1–3^ Experienced CHF symptoms, such as shortness of breath, fatigue, and limited physical functioning, may increase the illness workload, making completing tasks more difficult, which could be a contributor to poor treatment engagement.^4,5^ Patient engagement to self-management behaviors plays a key role in the effectiveness of CHF treatment and disease progression. Behaviors such as adherence to medication and physical activity have been shown to predict hospitalization, quality of life, and life expectancy.^6–8^

Nonadherence to treatment recommendations, such as medication adherence and physical activity, is present in many patient populations, including patients with CHF.^9–11^ Although interventions to support CHF self-management are promising, there is limited evidence on feasible yet cost-effective interventions and the “optimal education content, delivery method, intensity, and timing” of interventions.^6,12^ One self-management intervention that has shown particular promise in routine clinical care in the Netherlands is the Adherence Improving self-Management Strategy (AIMS-HIV) for patients with human immunodeficiency virus (HIV).^13,14^ The AIMS-HIV intervention is a theory-based (theory of planned behavior and control theory),^15,16^ nurse-delivered counseling intervention and has been shown to be feasible in routine clinical care, acceptable to patients, clinically effective (a reduction of 61% in the odds of treatment failure), and cost-saving.^13,14^ It includes nurse education to enhance knowledge and address misconceptions, motivational techniques focusing on patient attitudes and normative beliefs, problem-solving activities targeting self-efficacy and coping skills, and electronic medication monitoring and structured discussion of patients' medication intake to enhance behavioral awareness and self-regulation skills (ie, self-monitoring and problem solving).^13,14^ Hence, in AIMS, the constructs from the theory of planned behavior (which describes that attitudes, perceived social norms, and self-efficacy drive behavioral intentions, and self-efficacy and intentions drive behavior) are complemented with those from the control theory (which describes how people translate intentions into behavior: setting a goal, monitoring progress, and responding to difficulties by changing strategy or revising the goal), to overcome the gap between behavioral intentions and actual behavior (the so-called intention-behavior gap).^17^ The theoretical framework and intervention techniques of AIMS-HIV have been described in more detail elsewhere.^14,18^

Adherence Improving self-Management Strategy–HIV was initially piloted using a pre-post design and showed promising results in terms of feasibility, acceptability, and adherence. Next, a single-center randomized controlled trial was conducted with medication adherence as the primary outcome, which showed effects on medication adherence and clinical outcomes such as viral load. Finally, a multicenter randomized controlled trial was conducted with viral load as the primary outcome and cost-effectiveness modeling. This final study demonstrated clinical effectiveness (eg, 61% reduction in “treatment failure”) and cost-effectiveness of AIMS-HIV, which has consecutively been incorporated in HIV treatment guidelines.^14,19,20^

The question is whether this approach is useful beyond HIV. Wu and colleagues^21^ developed a CHF medication adherence intervention inspired by AIMS-HIV and similar interventions, and showed promising initial results for patients with CHF. Moreover, comparing HIV care with CHF care in the Netherlands, there are many similarities regarding the organization of care. For example, patients typically receive secondary care in hospitals, and nurses are primarily responsible for supporting self-management. In addition, self-management behaviors such as medication adherence are critical to treatment success.^6–8^ Therefore, rather than developing a new self-management intervention for CHF, we opted for adapting the AIMS-HIV intervention to CHF care (AIMS-CHF), recognizing however that CHF self-management may be more complex than for HIV.^22,23^

In this article, we report on the systematic, theory- and evidence-informed adaptation of AIMS-HIV to AIMS-CHF. The systematic description of the intervention development process, the choices made when moving from theory to practice, and the final intervention materials are critical to the advancement of behavior change science.^24,25^ The current article therefore describes these steps and decisions. Research is ongoing to determine whether this intervention is effective in patients with CHF (number in trial register: NCT04698954).

The specific research questions for the current study are the following:

1. What changes are required for adapting AIMS-HIV to AIMS-CHF in terms of the self-management behaviors (such as medication adherence and physical activity), behavioral determinants (such as self-efficacy, goal setting, and self-regulation), intervention methods (such as motivational and self-management techniques), and practical strategies (such as the written protocol, electronic monitors, and supporting visuals)?
2. Do patients with CHF and heart failure (HF) nurses perceive the AIMS-CHF to be acceptable and feasible in their context?

## Method

For the adaption of AIMS-HIV to AIMS-CHF, we used the intervention mapping adaptation framework, which is a systematic approach for adapting existing interventions to new populations and settings.^26^ In intervention mapping, it is also common to incorporate multiple theories to understand and change health behaviors, as is also the case in the AIMS intervention. In this article, we report on the four intervention mapping steps as summarized in Table 1. For this purpose, we analyzed the AIMS-HIV intervention materials, including the logic model of change, theoretical methods of change, practical applications, counseling protocol, and supporting visuals. Subsequently, we conducted a literature review on determinants of CHF self-management behaviors, organized advisory board meetings with HF nurses and patients with CHF, and consulted behavioral theories. Finally, we pretested key intervention components for feasibility before finalizing the AIMS-CHF protocol and supporting visuals.

**TABLE 1:** Intervention Mapping Steps for Adapting the Adherence Improving Self-management Strategy From Human Immunodeficiency Virus Care to Care for Patients With Chronic Heart Failure, Including the Methods Used

Step	Task	Methods Used/Source of Data
Step 1. Conduct needs assessment to compare the problem analysis underlying the original AIMS-HIV with a novel problem analysis underlying CHF self-management.	a. Create a logic model of the problem for the new at-risk group (CHF). b. Compare the logic model of the problem (CHF) with the original at-risk group (HIV) and note differences and similarities.	- Literature review - Healthcare provider input
Step 2. Performance objectives and change objectives: Examine how well the program objectives of AIMS-HIV fit with CHF-self-management (problem analysis, context) and modify where required.	a. Prepare or obtain a logic model of change of the original program (including behavioral and environmental outcomes, performance objectives, determinants, and change objectives). b. Prepare a logic model of change for the new setting. Add or delete objectives and/or determinants if needed.	- Literature review - Matrix of objectives (Tables 2 and 3)
Step 3. Theoretical methods and practical applications: Examine whether the original AIMS-HIV intervention methods and strategies fit with the AIMS-CHF (change objectives, context) and modify where required.	a. Review the original program and analyze the (theory-based) methods and practical applications. b. Make sure all change objectives are covered by appropriate and sufficient methods for the new program. c. Make sure all methods are adequately covered with practical applications.	- Advisory board meetings (6 HF nurses and 4 patients with CHF) - Taxonomy of behavior change methods - Table of selected behavioral change methods (Table 4)
Step 4. Program production: Adapt the AIMS-HIV program (intervention steps, content, visuals, tools) to fit with AIMS-CHF (change objectives, methods and strategies, context) and pretest critical program elements.	a. Make sure the theoretical methods match the scope, sequence, materials and delivery channels of the program. Determine integrity of the old and new program. b. Pretest the material and modify if needed.	- Literature review - Advisory board meetings (6 HF nurses and 4 patients with CHF) - Feasibility testing (9 HF nurses and 5 patients with CHF)

Abbreviations: AIMS, Adherence Improving self-Management Strategy; CHF, chronic heart failure; HF, heart failure; HIV, human immunodeficiency virus.

### Literature Review

The purpose of our literature review (search date: April 2019) was to identify the most important self-management behaviors in CHF and their behavioral determinants. The review was conducted in PubMed/MEDLINE, PsycINFO, and EMBASE (for search string, see Supplementary Table 1, http://links.lww.com/JCN/A280). Articles were included when adults (>18 years old) with CHF were studied, including at least 1 psychological determinant (eg, self-efficacy, beliefs, motivation) and at least 1 CHF-specific self-management behavior (eg, medication adherence, physical activity, general self-care), published in English, and available as full text. For efficiency, we first read literature review articles that synthesized quantitative and/or qualitative studies. In addition, single qualitative articles were examined to get a more in-depth understanding of the population with CHF, their beliefs, and barriers toward self-management.

### Advisory Board Panels

The purpose of the advisory board meetings was to engage the intervention providers (ie, nurses) and recipients (ie, patients with CHF) in the intervention adaptation decisions, to increase the likelihood that AIMS-CHF fits with daily clinical practice and patients' needs. Six HF nurses and nurse practitioners as well as 4 patients with CHF were recruited in 2 hospitals (1 academic and 1 regional) via convenience sampling. The 3 advisory board meetings with HF nurses lasted 2 to 3 hours per session, and the 2 advisory board meetings with patients with CHF lasted 3 to 4 hours per session. At each meeting, an overview over the AIMS-CHF intervention was presented, followed by a step-by-step discussion of the protocol and materials. Nurses and patients were invited to discuss the interventions' usefulness, completeness, necessity, relevance, correctness, comprehensibility, attractiveness, and acceptability. The researcher probed for further information and invited participants to respond to each other. Opinions of each member were gathered, notes were taken by a second researcher (A.M.J., B.M.A.v.B.), and audio was recorded as backup for later analysis. Advisory board input was processed in the next iteration of the materials and presented at a consecutive meeting, until the main concerns and suggestions were addressed (which was after 3 nurse meetings and 2 patient meetings).

### Feasibility Testing

The aim of pretesting essential components of the AIMS-CHF intervention was to determine the feasibility and acceptability of 3 key intervention components. First, we assessed the user experience (attractiveness, clarity, efficiency, controllability, motivation, and data output) of the electronic monitoring devices for medication adherence and physical activity in 5 patients with CHF.^27^ After 4 weeks of use, patient experiences were discussed through telephone interviews. Second, the comprehensibility and usefulness of discussing the data output from the electronic devices, including the process of downloading and presenting the behavioral data to patients, were evaluated with 9 HF nurses. Third, 9 HF nurses were trained in AIMS-CHF, after which all AIMS intervention steps were evaluated at the end of the training.

## Results

### Step 1: Needs Assessment

**FIGURE 1:**
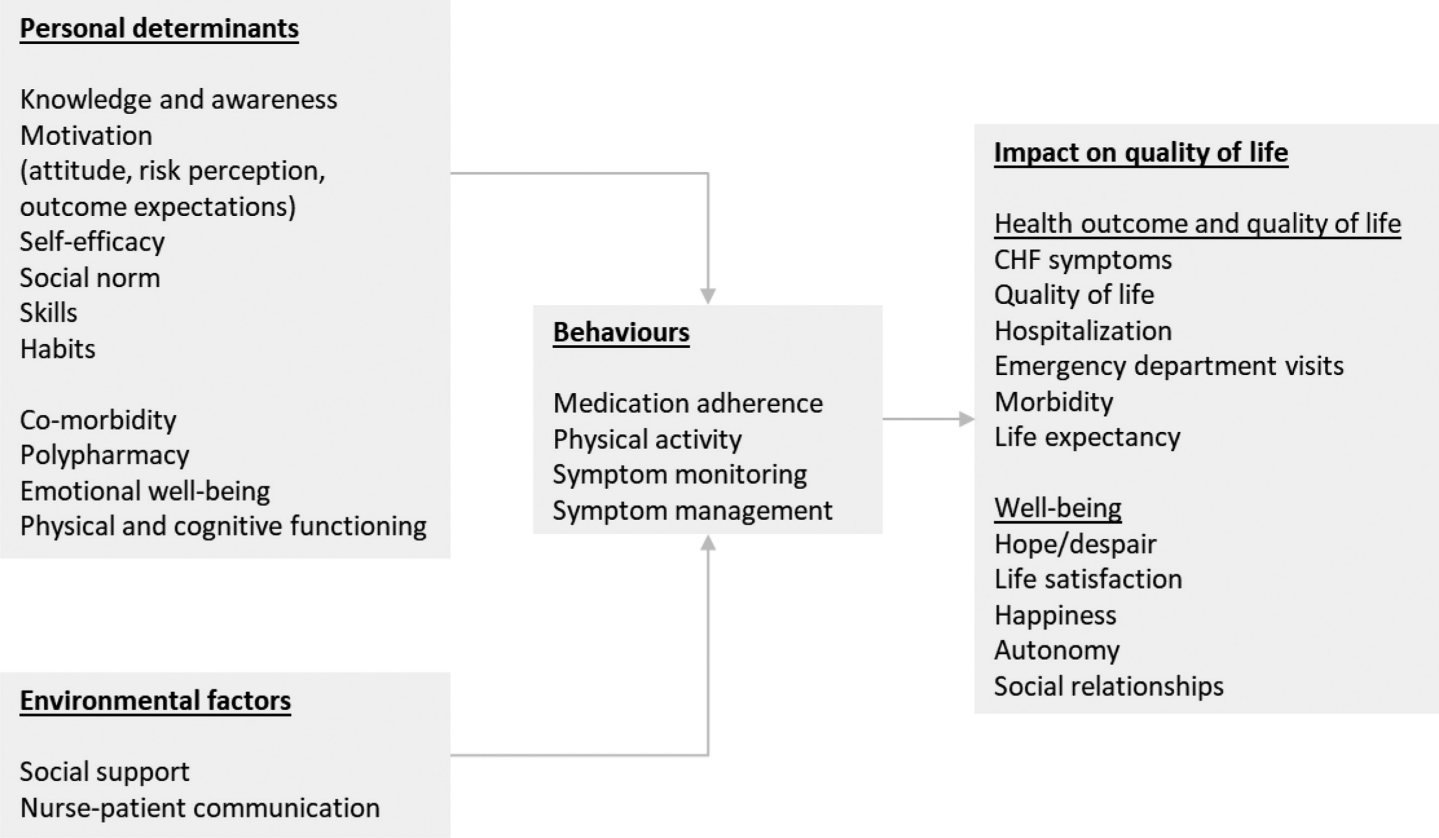
Logic model explaining chronic heart failure (CHF) self-management behaviors, based on the literature review and advisory board input.

The first step for adapting behavioral interventions is to conduct a needs assessment (as described in Table 1). This includes identifying the health-related outcomes of CHF self-management behaviors and the key determinants of these behaviors. The needs assessment is based on the literature review and advisory board meetings. It is summarized in the logic model in Figure 1, which is discussed hereinafter.

#### Self-management Behaviors and Their Impact on Outcomes

On the basis of the literature review, it is evident that the success of CHF treatment heavily relies on 4 self-management behaviors: medication adherence, physical activity, symptom monitoring, and timely healthcare seeking when symptoms occur/worsen.^22,23^ These self-management behaviors can lower the risk of all-cause mortality, alleviate symptoms, increase quality of life, and reduce hospital admissions.^8,28–31^ However, many patients struggle with implementing self-management behaviors in their daily life.^31^ First, medication nonadherence is associated with increased CHF exacerbations, reduced physical function, and higher risk of hospital admission and death.^8^ Second, the benefits of physical activity are well recognized, such as improved functional capacity, quality of life, and prognosis (European Society of Cardiology class IA recommendation).^32^ However, most studies show transient improvement in physical activity, for instance, during cardiac rehabilitation programs. Ultimately, most of the patients relapse into a physically inactive lifestyle.^33^ These findings underline the need for long-term guidance of physical activity promotion.^34^ Third, self-monitoring of symptoms helps to identify worsening symptoms at an early stage, so patients can seek timely help (fourth behavior), thereby preventing an acute symptom exacerbation. However, many patients experience difficulties in recognizing early subtle signs, resulting in delayed response.^35,36^ Hence, these 4 self-management behaviors are key to patient and healthcare outcomes, yet many patients seem to struggle performing or maintaining those behaviors.

After discussing the outcomes of the literature review with our advisory board panels, it was decided to focus AIMS-CHF on medication adherence and physical activity only. The rationale for that was that symptom monitoring and timely healthcare seeking were already adequately covered in patients' routine CHF care in the hospitals involved.

#### Understanding Adherence and Physical Activity in Patients With Chronic Heart Failure

Available literature clearly describes important factors (determinants) influencing medication adherence and physical activity. These can be grouped under nonmodifiable determinants, such as age and comorbidities, and modifiable factors, such as knowledge, motivation, and self-efficacy. Hereinafter, we present a brief summary of those determinants, whereas a more extensive summary can be found in Supplementary Table 2, http://links.lww.com/JCN/A280.

First, the most important nonmodifiable determinants (ie, factors that are too difficult or impossible to modify in the context of a nurse-delivered self-management intervention in routine clinical care) identified for CHF self-management were emotional well-being,^37–39^ cognitive impairment,^37,38,40–42^ comorbidity,^1,38,43,44^ and consequent polypharmacy.^45,46^ Comorbidity and polypharmacy are common in patients with CHF and increase the complexity of the medication regimen.^1^ Contradicting advice and multiple changes in medication regimen (switch of medication type or dose; changes in pill size, color, or shape)^44,45^ can also lead to confusion.^38,46^ Comorbidity is accompanied by the importance of engaging in multiple behaviors and keeping an overview of complex treatment plans, which is often challenging for patients.^38,43,46^ Although these determinants of CHF self-management cannot be altered in this nurse-counseling intervention, they need to be taken into account when developing the intervention strategy and materials.

Second, multiple modifiable determinants have been found to be related to adherence and physical activity. First, patient knowledge and awareness regarding the cause of CHF, its progressive nature, and the role of self-management behaviors such as medication adherence and physical activity are often suboptimal.^1,22,47–49^ Although knowledge is necessary, it is in itself insufficient for behavior change.^50^ Other determinants such as motivation, self-efficacy, and skills are also important. Important treatment motives for patients with CHF that have been identified are maintaining autonomy, physical well-being, maintaining social relationships, and symptom relief.^1,22^ Self-efficacy, patients' confidence in their ability to self-manage CHF, is also consistently and strongly associated with CHF self-management.^1,23,37,51^ High self-efficacy facilitates setting challenging goals, sticking to them even under challenging circumstances, and learning from previous success or failure.^1,49^ Self-management skills such as setting goals, action planning, and making suitable decisions are also important in CHF self-management.^22,49,52^ When patients get used to the medication regimen, the creation of daily routines and, eventually, habits could facilitate CHF self-management in the long term, but patients may struggle creating those routines.^1,22,48^

The literature reveals 2 relevant modifiable environmental factors, namely, nurse communication and social support from family and friends. Technical medical or inconsistent language and poor communication skills complicate understanding of instructions and may cause confusion, loss of interest, and gaps in patient knowledge among patients with CHF.^1,35,38^ On the contrary, confidence in the nurse and a good patient-nurse relationship are important conditions for effective self-management support.^1,38^ Social support from family member(s) and other relatives is also influential. All 4 types of support (ie, emotional support, instrumental/tangible support, informational support, and appraisal support) seem important, although it is unclear which type is most beneficial.^53^

Taken together, interventions to support CHF patient self-management are likely to be more effective if they successfully address this range of individual and environmental determinants. It is relevant to note that the determinants targeted by the original AIMS-HIV intervention are very similar to these modifiable determinants of CHF self-management.^18^

### Step 2: Performance Objectives and Change Objectives

In step 2 of intervention mapping, the results from the needs assessment (step 1 above) are used to formulate concrete intervention objectives. For this purpose, the following specific behaviors (ie, performance objectives) were formulated: (1) initiating behavior change, (2) maintaining behavior change, and (3) improving communication with the healthcare provider (see column 1 of Table 2). The combination of these objectives with the most relevant modifiable determinants (depicted in row 1 of Table 2) allows for formulating specific change objectives depicted in the cells where performance and change objectives cross. These change objectives indicate exactly what a patient would need to develop or learn from the intervention to change their behavior. A similar matrix was developed for physical activity, but for brevity, this is included in Supplementary Table 3, http://links.lww.com/JCN/A280.

**TABLE 2:** A Matrix of Change Objectives for Chronic Heart Failure Medication Management

Performance Objectives	Knowledge/Awareness	Risk Perception	Attitude/Outcome Expectation	Self-efficacy/Skills/Habit	Social Norm/Social Support
PO.1.1. Take medication correctly (right dose, time, interval) and consistently (execution)	K.1.1.a. The patient can recall or is able to retrieve prescribed medication regarding the following aspects: *what*: active substance + doses, *when*: time and interval. K.1.1.b. The patient can describe the role of medication adherence in relation to treatment success and the chronic condition. K.1.1.c. The patient can recall what to do, when missing a dose.		A.1.1.a. The patient expresses positive feelings about the benefits of medical treatment; there are no undisputed concerns regarding the treatment. A.1.1.b. The patient sets a concrete medication adherence goal that he/she wishes to achieve and is able to explain why this is important for him/her. A.1.1.c. The patient can explain the discrepancy between desired medication intake and current medication intake, and is willing to bridge the gap.	SS.1.1.a. The patient demonstrates the ability of monitoring and evaluating his/her own medication adherence behavior. SS.1.1.b. The patient demonstrates the ability of recognizing barriers of correct and consistent medication intake and identify effective solutions. SS.1.1.c. The patient demonstrates the ability of dealing with/anticipating to (unforeseen) barriers and is able to adapt to new situations. SS.1.1.d. The patient express confidence in achieving his/her personal medication adherence goal and is able to organize the medication in a personally effective way (matching daily life).	SN.1.1.a. The patient feels supported by his/her social environment (including HF nurse) regarding correct and consistent medication intake, and recognizes that asking for help is essential in the treatment of CHF. SN.1.1.b. The patient recognizes that there are different types of support, knows what type of support is personally desirable, and is able to express this.
PO.1.2. Continue with lifelong medication treatment (maintenance)		R.1.2.a. The patient experiences CHF as a chronic/progressive disease and is aware of the importance of maintaining medical therapy for the short as well as the long term, related to personal values and life goals.		SS.1.2.a. The patient is confident in his/her ability to maintain correct and consistent intake of the medication, to monitor this and has effective strategies to deal with barriers and challenging situations. SS.1.2.b. The patient attributes success as stable and internal, and failure as controllable and a learning experience.	SN.1.2.a. The patient feels supported by his/her social environment (including HF nurse) regarding long-term medication intake and recognizes that asking for help is essential in the treatment of CHF. SN.1.2.b. The patient recognizes that there are different types of support, knows what type of support is personally desirable, and is able to express this.
PO.1.3. Open and timely communication with HF nurse	K.1.3.a. The patient knows how and when to ask the HF nurse for help.		A.1.3.a. The patient finds it important to share concerns/difficulties, asks questions, and asks timely for help.	SS.1.3.a. The patient feels capable of asking questions, sharing difficulties, and asking timely for help.	

When applying a modifiable determinant (eg, knowledge) to a specific performance objective (taking medication correctly and consistently), the resulting change objective reflects what the person exactly needs to learn or change as a result of the intervention.

Abbreviations: CHF, chronic heart failure; HF, heart failure.

### Steps 3 and 4: Selecting Behavior Change Methods and Developing Program Materials

Intervention mapping steps 3 and 4 focus on developing an intervention program and materials that fit with the clinical context (ie, nurses delivering AIMS-CHF during routine care) and target group of patients with CHF and should contribute to achieve the change objectives described in Table 2.

#### Selecting Behavior Change Methods

In step 3 of intervention mapping, behavior change methods are identified that are suitable for changing the determinants described in the matrices (step 2).^26^ Intervention mapping contains a taxonomy of behavior change methods designed for this purpose.^54^ For example, the behavior change method “consciousness raising” is defined as “delivering information, feedback or confrontation about cause, consequence, and alternatives for a problem” and can affect someone's risk awareness and motivation to change. It is important though that raising risk awareness is quickly followed by strategies for increasing a person's self-efficacy or problem-solving skills; otherwise, this method is ineffective or may even backfire (eg, disengagement). The intervention mapping taxonomy describes numerous behavior change methods and provides guidance on how these should be applied in intervention for optimal effectiveness.

Given the similarities in behavioral determinants between the original AIMS-HIV and AIMS-CHF, we were able to use the behavior change methods from AIMS-HIV as the backbone for AIMS-CHF as well (Supplementary Table 4, http://links.lww.com/JCN/A280, provides an overview of behavior change methods and their definitions included in AIMS-CHF).

#### Developing the Program Materials

Although the determinants of HIV and CHF self-management behaviors, as well as the behavior change methods selected, were very similar for AIMS-HIV and AIMS-CHF, the practical intervention materials (counseling protocol, patient information, graphics) required numerous adaptations to modify AIMS-HIV to AIMS-CHF. These adaptations involved regular and iterative feedback from patients with CHF and HF nurses, which is described hereinafter in more detail and illustrated in Figures 3 and 4.

#### Defining the Scope and Sequence of Adherence Improving self-Management Strategy–Chronic Heart Failure

**FIGURE 2:**
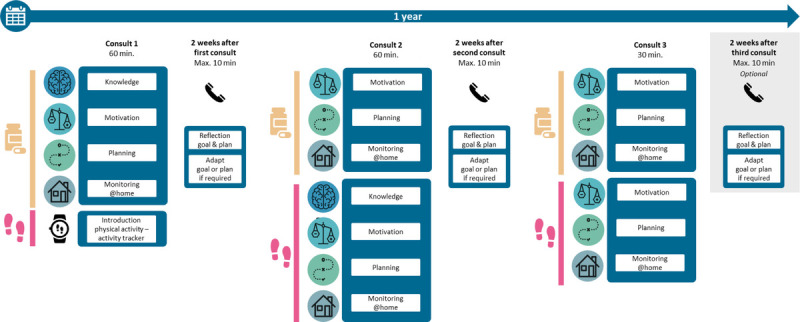
An overview of key intervention activities and timeline over a 12-month period, composed of 3 face-to-face consultations and follow-up phone calls, and electronic monitoring of behaviors. The content of each visit is indicated for both behaviors (medication and steps symbols) and related content (text boxes).

Adherence Improving self-Management Strategy–CHF focuses on supporting both medication adherence and physical activity, delivered by trained HF nurses during routine clinic visits. Key activities include providing knowledge, discussing motivation, action and coping planning, electronic self-monitoring, feedback, and reinforcement. These activities demand an active participation of the patient. The patient advisory board recommended to introduce medication adherence and physical activity in consecutive visits, to prevent information overload and develop a routine for one behavior before starting on the next. It was known from AIMS-HIV, which focuses on medication adherence only, that the first intervention visit takes twice as long than follow-up visits.^13^ The nurse advisory boards decided that the first 2 sessions (the first focusing primarily on adherence and the second on physical activity) had to be extended to 60 minutes instead of the usual 30 minutes. The third intervention session would then focus on establishing long-term habits for both behaviors. In addition, a follow-up phone call was incorporated 2 weeks after the first 2 visits to answer any questions and make early adjustments to personalized plans and goals if necessary. Hence, the input of the advisory boards was key in this stage and resulted in what should be a more feasible and effective intervention strategy, as summarized in Figure 2 hereinafter.

#### Developing and Producing the Final Program Materials

The program materials were composed of a counseling protocol, easy-to-remember graphics to discuss more complex information, and electronic monitoring tools and adherence and physical activity feedback reports. For this, the counseling protocol of AIMS-HIV was rewritten and redesigned to fit with CHF adherence and expanded to also include the physical activity protocol. Visuals were designed for each counseling step to support both patients and also nurses to enhance intervention fidelity to the counseling protocol.

**FIGURE 3:**
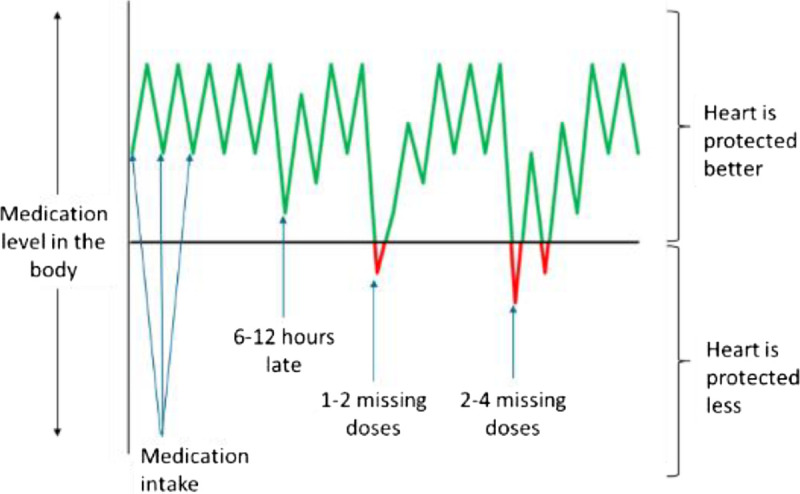
This figure is used by heart failure nurses to discuss with patients the rationale of regular and consistent intake of medication. Nurses explain that when patients take the medication regularly and in the right dose, their medication levels are in the green zone (heart is protected better) and that (regular) missed doses could potentially lead to added damage and complications (red zone). Nurses prompt patients to ask questions and discuss concerns. The curve above the horizontal line is colored green and below colored red.

**FIGURE 4:**
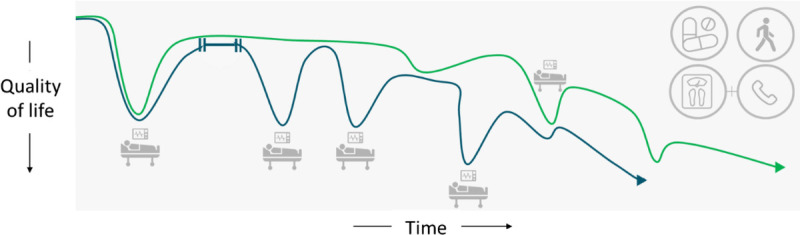
This figure is used by heart failure nurses to discuss with patients the benefits of treatment and self-management. The nurse discusses the benefits of following treatment and self-management recommendations, by discussing the differences between a typical course of illness without effective (lower line) and with effective treatment and self-management (higher line). It is used to illustrate the likely effects on hospitalizations, quality of life, and life expectancy.

The quotes hereinafter illustrate the type of input from the advisory boards. For example, the nurse advisory board approved the graphics developed for AIMS-CHF (eg, “It [see Figure 3] is really imaginative and intuitive, I like it and see myself using it” [nurses]) but also suggested several content and visual modifications. A good example of the discussion and of the importance of tailoring the AIMS intervention to individual patients is reflected in the quotes hereinafter regarding Figure 4.

For me this information and image is valuable, if the health care professional will not provide me such information, I will ask Google. (patient)

This kind of information is difficult to present at a strategically good time or moment, it is confrontational and could be helpful when you are ready for it, but could also have an opposite effect [scare someone]. (patient)

I find the graph too confrontational, due to the red line which is too negative, the green line which is too positive, and both lines ending at the bottom corresponding to death. (nurse)

We do not agree. Patients do need to know CHF is a serious illness and need to face reality. We say: “Hope for the best and prepare for the worst. (2 nurses)

#### Electronic Monitoring Devices and Feasibility Testing

Adherence Improving self-Management Strategy–CHF uses an electronic medication monitor and physical activity tracker as part of the intervention. Electronic monitors were selected based on preset criteria (see Supplementary Table 5, http://links.lww.com/JCN/A280). For medication adherence, the medication adherence button (MEMS; Aardex Group, Liege, Belgium) was selected. For physical activity, the Garmin Vivofit 4 (activity tracker) was deemed most suitable, also for its validity in assessing walking characteristics in elderly populations.^55^ After a pretesting period of 4 weeks, 6 patients with CHF rated the medication button as easy to use and not burdensome, although some patients complained that it lacked direct feedback (eg, seeing the date and time of the last medication intake). Downloading the data from the medication button was experienced to be easy by HF nurses, and the resulting reports were found useful in assessing one's own adherence patterns over longer periods.

Furthermore, the Garmin activity tracker was easy to use and motivating because it offered both direct and delayed feedback. Critical notes were given regarding some functions and its controllability. For example, 2 patients stated that the cue for inactivity (sound and vibration) was motivating at first but, after a while, irritating. Downloading the data from the Garmin was more time consuming and complex. As a backup, HF nurses required an instruction manual for downloading the Garmin data.

### The Final Intervention Protocol and Materials

The 4-step process of intervention adaptation—using the original AIMS-HIV intervention, a literature review, behavioral theory and taxonomy, and extensive advisory board input—all accumulated into a blended (nurse counseling, graphics, electronic monitoring, and feedback) AIMS-CHF intervention protocol in which all change objectives (from Table 2) and behavior change methods (from Supplementary Table 4, http://links.lww.com/JCN/A280) were applied. Supplemental Table 6, http://links.lww.com/JCN/A280, contains a full description of the first AIMS-CHF intervention session that shows all tasks, graphics, change objectives, and behavior change methods applied in each step. The full intervention protocol, materials, and training can be obtained from the corresponding author on request.

## Discussion and Conclusion

This article aimed to transparently describe the systematic development and adaptation of a multiple-behavior intervention for patients with CHF. We followed the 4 steps of the intervention mapping framework and used scientific literature, behavioral theory, and patient and HF nurse advisory boards throughout the process. We found that the drivers of self-management and the context (eg, nurse delivered, similar duration of consultations) were largely comparable with the original AIMS-HIV intervention, so most of the original objectives, methods, protocol, and materials could be retained. Most adaptations had to be made to the content and layout of AIMS materials and, of course, the extension of the intervention to also include physical activity. Completing all intervention mapping steps resulted—according to the advisory board and feasibility testing—in a feasible and acceptable theory- and evidence-based self-management intervention for patients with CHF in secondary care settings.

The main strength of this study is that we started working from an existing feasible, acceptable, and effective intervention and used a systematic approach and involved patients and HF nurses early and iteratively to adapt this to another clinical population. The intervention mapping adaptation framework forces researchers to think thoroughly of all the important steps in the adaptation process, with the only downside that this process can be quite time consuming.^26^ We therefore chose to consult existing systematic reviews, qualitative studies, and advisory boards to get a sound understanding of important determinants of CHF self-management and their context, rather than conducting a systematic review and a qualitative study ourselves. For a close alignment with (clinical) practice and needs of people involved, it is also a prerequisite to involve end users. The advisory board meetings allowed us to iteratively incorporate feedback of the end users (ie, patients with CHF and HF nurses) and come to an acceptable, feasible, and tailored intervention.

One of the biggest challenges in the adaptation process of interventions is the necessary deconstruction of the intentions of the original program developer.^56^ A lack of communication with the original developer and/or a lack of a comprehensive intervention description, including theoretical foundations and rationale, can hinder intervention adaptation.^56,57^ In this study, the developer of AIMS-HIV (author M.d.B.) was closely involved in the intervention adaptation process.

The purpose of publishing intervention development and adaptation articles is so that people can learn from how others have developed their interventions.^24^ Moreover, for cumulative science, it is essential that interventions are fully described so that others can replicate or adapt them and can adequately synthesize the available evidence.^25,58^ We therefore aimed to transparently report on the complete adaptation process, show the results of each intervention mapping step, and present the final intervention materials.^57^ As the adaptation of health behavior change interventions to other populations or conditions is uncommon, we hope that this article offers a useful illustration of how that can be done systematically.

This study also has several limitations. First, we opted for a one-size-fits-all electronic medication button to assess medication adherence, whereas a variety of medication packaging options (eg, a variety of strips, Baxter packing or medication bottles) were used in daily practice. A limitation is that this button requires an additional action from the patient to register medication intake (press a button) rather than that it automatically registers patients accessing their medication packaging. If patients forget to press the button or get tired of doing so, this would lead to underestimating patients' true adherence. The physical activity monitor (Garmin) is a commercially available activity tracker, but the software is not designed for clinical use. We do foresee that not all patients will be able to install and use the smartphone application independently, so that additional support is likely to be necessary. Hence, although the integration of eHealth into medical care is increasing, the hardware and software solutions for monitoring and feedback of health behaviors are far from ideal yet. A third limitation of the study is that our advisory boards represent a selection of motivated and engaged nurses and patients. Hence, in future pilot and effectiveness studies, it will be important to conduct process evaluations to further fine-tune AIMS-CHF to patients and care providers' needs and abilities.

## Conclusion

This study shows that AIMS-HIV, an (cost) effective nurse-delivered intervention to promote self-management of medication adherence, can be adapted to CHF and expanded with additional self-managements behaviors. It was found to be acceptable and feasible by patients as well as HF nurses. The intervention mapping adaptation framework is valuable as a guidance, although the process is still time consuming. The early and iterative involvement of the intervention target group (patients with CHF) and HF nurses was crucial in this process. Although AIMS-HIV has shown to be clinically and cost-effective for HIV in previous research, its (cost) effectiveness in CHF care will have to be determined in a future trial (initial effectiveness study ongoing, number in trial register: NCT04698954).

**What’s New and Important**

1. We show how intervention mapping can be used to systematically adapt an (cost) effective patient self-management intervention from one condition and patient population (HIV) to another (CHF).
2. Important behavior change methods and mechanisms of action of patient self-management interventions in one clinical domain can—with some modifications—be used as the basis of self-management interventions in another clinical domain, benefiting transferability of evidence and skills in nursing science and practice.
3. This methodology transparently shows how multiple-behavior change methods targeting key drivers of self-management can be efficiently combined in a blended (nurse counseling, visuals, technology) intervention that trained nurses should be able to deliver in routine care.
